# Treatment of Dry Ears with Partial Deafness

**Published:** 1834

**Authors:** 


6. *Treatment of dry Ears, with partial Deafness.*—Dr. W. M. Coates, (*Bost. Journal,*) has some practical observations, on a common but perplexing form of disease in the meatus externus, which are worthy of attention. After speaking of a deficient secretion of the *cerumen* of the ear from a common cold, which he remarks is removable by a pediluvium and a warm bed, he goes on to say—

"But there is a dry state of this part strictly local, and which has always appeared to me to have arisen from an indolent state of the ceruminous glands. The symptoms are more or less deafness—sometimes to a great degree. When the patient happens to catch a sound, it is sharp, and causes unpleasant noise in the ears for some time after.

On examining the part the cubicular lining is white, and quite destitute of the ordinary secretion. The following case illustrates pretty well the affection, and the mode of treatment which I have found most successful:—

"Charlotte Osborne, aged 22, has been deaf nine months, the complaint having gradually arrived at its present intensity. Present symptoms, May 14, 1833: very deaf of both ears, but more particularly so of the right. Of a costive habit; tongue white and dry; of heavy aspect, and inattentive behaviour. Left ear quite destitute of cerumen; the right has rather more than its usual proportion. Her ears were syringed with soft soap and moderately warm water; and she was ordered—Pilulæ Hydrargyri, gr. iij. omni nocte. Magnesiæ Subcarbonatis, gr. viij.; Rhei Pulveris, gr. x. omni mane.

May 19th.—Tongue clean and moist; bowels freely open. The left ear is evidently worse; the right is improved somewhat. Having syringed both ears, I applied, by means of a small camel's hair brush, to the left membrana tympani, a very minute portion of the following ointment, rendered soft by the admixture of a little almond oil. Unguenti Hydrargyri Nitratis Diluti, ʒj.; Unguenti Cetacei ʒiij. Misce.

21st.—Has had some slight pain in the left ear, but can hear much better with it; the right continues to improve. The ointment to be applied to the left membrana tympani every other morning.

July 28th.—She has continued this treatment ever since. She has become of ruddy complexion, lusty, and lively in her behavior. She expresses herself as hearing as well as she ever did.

I must observe, that the cure was retarded considerably by a severe cold caught during the latter part of the treatment."

The following affection, although less frequent than the preceding, is more obstinate.

"There s another species of deafness, differing in its cause from the above, but very often mistaken for it. It arises from a faulty secretion from the ceruminous glands, and occurs now and then with, or alternating with, strumous ophthalmia. In these cases there is a good deal of irritation and noise in the ears. On examination of the external meatus there is no deficiency, but occasionally an unnatural dingy color of the cerumen. This affection occurs ordinarily in persons accustomed to unwholesome food, and destitute of the common comforts of life. As the causes can seldom be removed, we treat the disease under very unfavorable circumstances; however, I have generally succeeded in curing this affection for the time being, but on the patient returning to his old habits, like all scrofulous complaints (for I believe this to be almost always dependent on a scrofulous diathesis,) it generally re-appears.

The first indication is to correct the secretions from the bowels, which are generally of an unnatural character, by mild mercurials; I generally use the hydr. c. creta in small and repeated doses. The second, to diminish the irritability of the secreting membrane; this I do by the application of a solution of the argenti nitras, in the proportion of one grain to the ounce of distilled water.

